# Relative impact of pre-eclampsia on birth weight in a low resource setting: A prospective cohort study

**DOI:** 10.1016/j.preghy.2020.04.002

**Published:** 2020-07

**Authors:** Annettee Nakimuli, Jennifer E. Starling, Sarah Nakubulwa, Imelda Namagembe, Musa Sekikubo, Eve Nakabembe, James G. Scott, Ashley Moffett, Catherine E Aiken

**Affiliations:** a Department of Obstetrics and Gynaecology, Makerere University and Mulago National Referral Hospital, Kampala, Uganda; b Red McCombs School of Business and Department of Statistics and Data Sciences, University of Texas at Austin, TX, USA; c Department of Pathology and Centre for Trophoblast Research, University of Cambridge, Cambridge, United Kingdom; d Department of Obstetrics and Gynaecology, University of Cambridge, Box 223, The Rosie Hospital and NIHR Cambridge Biomedical Research Centre, Cambridge CB2 0SW, United Kingdom

**Keywords:** Pregnancy, Pre-eclampsia, Birth-weight, Sub-Saharan Africa, Uganda, Gestational age

## Abstract

**Objectives:**

Low birth-weight is a major risk factor for perinatal death in sub-Saharan Africa, but the relative contribution of determinants of birth-weight are difficult to disentangle in low resource settings. We sought to delineate the relationship between birth-weight and maternal pre-eclampsia across gestation in a low-resource obstetric setting.

**Study design:**

Prospective cohort study in a tertiary referral centre in urban Uganda, including 971 pre-eclampsia cases and 1461 control pregnancies between 28 and 42 weeks gestation.

**Main outcome measures:**

Nonlinear modeling of birth-weight versus maternal pre-eclampsia status across gestation. Models were adjusted for maternal-fetal characteristics including maternal age, parity, HIV status, and socio-economic status. Propensity score matching was used to control for the severity of pre-eclampsia at different gestational ages.

**Results:**

Mean birth-weight for pre-eclampsia cases was 2.48 kg (±0.81SD) compared to 3.06 kg (±0.46SD) for controls (p < 0.001). At 28 weeks, the mean birth-weight difference between pre-eclampsia cases and controls was 0.58 kg (p < 0.05), narrowing to 0.17 kg at 39 weeks (p < 0.01). Controlling for pre-eclampsia severity only partially explained this gestational difference in mean birth-weight between pre-eclampsia cases and controls. Holding gestational age constant, pre-eclampsia status predicted 7.1–10.5% of total variation in birth-weight, compared to 0.05–0.7% for all other maternal-fetal characteristics combined.

**Conclusions:**

Pre-eclampsia is the dominant predictor of birth-weight in low-resource settings and hence likely to heavily influence perinatal survival. The impact of pre-eclampsia on birth-weight is smaller with advancing gestational age, a difference that is not fully explained by controlling for pre-eclampsia severity.

## Highlights

• Pre-eclampsia is the major determinant of birthweight across all gestations in Uganda.
• Pre-eclampsia accounts for ×10 more birthweight variability than all other factors.
• In pre-eclampsia, gestation predicts birthweight better than disease severity.

## 1 Introduction

Neonatal survival depends on a wide range of factors, but birth weight is a key determinant [1]. Globally, across all contexts, babies born at lower birth weights have a higher risk of perinatal death than babies who are appropriately grown for gestational age [2], [3], [4]. Tackling the high rates of death among babies born at low birth weights in low or middle human development index (LM HDI) countries relies on developing better understanding of the key risk factors in different populations.

In sub-Saharan Africa, pre-eclampsia is a common, severe, under-recognised, and under-treated maternal condition [5], [6], [7] that has the potential to influence birth weight. Genetic factors, poor baseline health status, and lack of access to high quality antenatal care [6] may all contribute to the severity of pre-eclampsia observed in sub-Saharan African women [5]. The perinatal death rate in pregnancies affected by severe pre-eclampsia or eclampsia in urban Uganda is two-fold higher than in normotensive women, [8] with some evidence suggesting a perinatal death rate of over 20% in pregnancies complicated by pre-eclampsia [9].

As a largely placental disease, pre-eclampsia is a recognized risk factor for low birth weight [10]. The initiating event in pre-eclampsia is incomplete conversion of the spiral arteries in early pregnancy, due to inadequate invasion of the vessel walls by the extra-villous trophoblast [11]. For the fetus, poorly formed materno-fetal vascular connections mean a relative lack of nutrients and hence restricted growth [12].

We sought to fully delineate the influence of pre-eclampsia on birth weight across viable gestations, and compare this to the influence of other maternal and fetal factors.

## 2 Methods

### 2.1 Study population

We conducted a prospective cohort study at Mulago National Referral and Teaching Hospital in Kampala, Uganda, which acts a tertiary referral centre for Uganda. Women were recruited to the study in three waves: July 2009, August 2010–June 2011, and September 2014-Dec 2016 (Fig. S1). There was no influence of wave or year of delivery on any of the modelling results. Mulago Hospital accommodates ~30,000 deliveries each year, making it one of the largest obstetric centres globally. Both women with pre-eclampsia and normotensive women were consecutively recruited from the maternity unit during each wave of the study. Data were collected by research midwives at the time of initial presentation using a researcher-administered questionnaire, and further information was abstracted from the medical record.

### 2.2 Outcome measures

The primary study outcome was birth weight, measured in kilograms. We also converted the birth weights observed in our population into gestational age and sex-adjusted centiles using an international reference standard [13]. Pregnancies were classified by presence or absence of pre-eclampsia. On recruitment into the study, women were designated by reference to the clinical notes as affected by pre-eclampsia (hereafter the “pre-eclampsia group”) or not affected by pre-eclampsia (hereafter the “normotensive group”). The pre-eclampsia status of every recruited woman was checked against diagnostic criteria, which were based on a context-appropriate adaptation of the ACOG Task Force Report on Hypertension in Pregnancy [14]. Pre-eclampsia was classified where systolic blood pressure was measured as ≥140 or diastolic as ≥90 on at least two occasions four hours apart, in conjunction with either ≥+1 proteinuria on dipstick or clinical seizure activity. In common with other low-resource settings [15], routine blood tests are not performed on all women presenting with hypertension and proteinuria in the study centre. Thus we were unable to apply the ACOG criteria that rely on laboratory tests. All women included in the analysis were concordant in their pre-eclampsia status from their contemporaneous clinical notes and assessment by the modified ACOG criteria. Women with known chronic hypertension or renal disease were excluded from the analysis. Severity of pre-eclampsia was assessed using the maximum systolic and diastolic blood pressures measured during the delivery episode, and maximum proteinuria level on dipstick.

Gestational age in weeks was calculated for each pregnancy based on last menstrual period or ultrasound scan. We considered only singleton infants born between 28 and 43 weeks estimated gestation. Infant sex was designated as male or female at delivery. We included ‘fresh’ stillborn babies (those judged by the clinician to have died shortly before delivery), but not ‘macerated’ stillbirths. We also included cases of early neonatal death.

Maternal-fetal characteristics for each pregnancy were recorded in the study data set, and were either derived directly from the clinical notes or from information provided by the women themselves. Maternal characteristics included maternal age (in years), ethnicity, and parity. Detailed information on the mother’s ethnicity and the family ethnicity (father and all four grandparents) was recorded, and encoded in our regression models as a binary variable for whether the mother belonged to the predominant local Ganda ethnicity. Women were asked whether they had experienced a severe febrile illness during the pregnancy. Given knowledge of local infectious disease prevalence and population susceptibility [16], this was likely to represent malaria during pregnancy (Appendix S1). We also obtained data on whether the mother was known to be HIV positive or not. Maternal occupation was self-reported by women in their own words and then classified by the research team using the ISCO-08 classification [17]. This classification was then further collapsed to three categories for the purpose of regression modelling: ‘professional’, ‘skilled’ or ‘unskilled/no occupation’.

### 2.3 Statistical analysis

In our primary analysis, we assessed the relationship between pre-eclampsia and birth weight across gestation. Regression splines were used to fit birth weight as a nonlinear function of both gestational age and pre-eclampsia status. To isolate the effect of pre-eclampsia, our model also adjusted for several other maternal-fetal characteristics, including stillbirth, infant sex, maternal age, maternal job type, parity, and HIV status. The model—including the location of spline knots and the variables/interactions included—was chosen using a stepwise selection process that is detailed in Appendix S2. Maternal ethnicity and presence of febrile illness during pregnancy were considered for inclusion in the model, but were discarded by the stepwise selection process, indicating that they were not significant predictors of birth weight.

Our spline model is highly flexible, in that it allows for a time-varying effect of pre-eclampsia on birth weight, i.e. an effect that varies continuously as a function of gestational age. Therefore, to calculate a model-adjusted average effect of pre-eclampsia across multiple gestational ages, we calculated Friedman’s partial-dependence function [18] for the pre-eclampsia variable. This is a standard measure of association in non-linear models. Specifically, we generated two model predictions for each patient in the dataset: one assuming that pre-eclampsia was present, and one assuming that pre-eclampsia was absent. The differences in these predictions can be interpreted as the patient-specific effect of pre-eclampsia on birth weight. We then averaged these patient-level differences, both across all gestational ages and within specific gestational age categories (<34 weeks, 34–36 weeks, and >36 weeks). We also conducted an analysis of variance (ANOVA) on our final model in order to quantify the percentage of variation in birthweight explained by pre-eclampsia versus other covariates, both across all gestational ages and separately by gestational age category. For details, see Appendix S3.

As a secondary analysis, we investigated whether a time-varying effect of pre-eclampsia on birth weight could be explained by the fact that cases of pre-eclampsia tended to be more severe at earlier gestational ages. To do so, we used propensity score matching to pair observations at high and low gestational ages with similar pre-eclampsia severity markers (Fig. S5). We then fit a spline model of the same form as in our primary analysis, but on a dataset including only those pre-eclampsia case that were part of a severity-matched pair. This approach includes all women in the normotensive group, but excludes pre-eclampsia cases that cannot be matched to a case of similar severity, but in the opposite (high or low) gestational age category. For further details on the model selection process and subsequent analyses, see Appendix S2–4 and Fig. S2. All data analyses were conducted using the R statistical software package version 3.3.4 [19]. The study was approved by Makerere University’s Faculty of Medicine Research and Ethics Committee (Reference numbers 2009-083 and 2014-065). All participants gave informed consent.

## 3 Results

2387 women were recruited to the study, of whom 971 had confirmed pre-eclampsia and 1416 were normotensive. The cohort characteristics are described in Table 1. Women with pre-eclampsia were more likely than normotensive women to be multiparous (p < 0.001), to have higher systolic (p < 0.001) and diastolic (p < 0.001) blood pressures, and to have higher proteinuria levels (p < 0.001). Women with pre-eclampsia were older (p < 0.001) and were more likely to experience stillbirth (p < 0.001). There was no difference between the pre-eclampsia and normotensive groups in terms of infant sex, HIV status, ethnicity, or likelihood of having experienced febrile illness during pregnancy.

**Table 1:** Maternal and fetal characteristics of pregnancies included in the study.

Characteristic		All (N = 2387)	No pre-eclampsia (N = 1416)	Pre-eclampsia (N = 971)	p-value
Gestational age (wks)	28–31	117 (4.90)	16 (1.13)	101 (10.40)	<0.001
	32–36	376 (15.75)	65 (4.59)	311 (32.03)	
	37–38	622 (26.06)	397 (28.04)	225 (23.17)	
	39–40	869 (36.41)	626 (44.21)	243 (25.03)	
	41–43	403 (16.88)	312 (22.03)	91 (9.37)	
Infant Sex	Male	1173 (49.14)	702 (49.58)	471 (48.51)	0.64
	Female	1214 (50.86)	714 (50.42)	500 (51.49)	
Maternal age (years):	<20	602 (25.22)	470 (33.19)	132 (13.59)	<0.001
	20–29	1107 (46.38)	623 (44.00)	484 (49.85)	
	30–39	209 (8.76)	42 (2.97)	167 (17.20)	
	≤40	10 (0.42)	1 (0.07)	9 (0.93)	
Maternal job type	Professional	827 (34.65)	450 (31.78)	377 (38.83)	<0.001
	Skilled	1195 (50.06)	765 (54.03)	430 (44.28)	
	Unskilled/none	365 (15.29)	201 (14.19)	164 (16.89)	
Stillbirth	No	2174 (91.08)	1326 (93.64)	848 (87.33)	<0.001
	Yes	213 (8.92)	90 (6.36)	123 (12.67)	
Parity	Primiparous	1840 (77.08)	1364 (96.33)	476 (49.02)	<0.001
	Multiparous	547 (22.92)	52 (3.67)	495 (50.98)	
HIV	No	2260 (94.68)	1340 (94.63)	920 (94.75)	0.98
	Yes	127 (5.32)	76 (5.37)	51 (5.25)	
Blood pressure	Systolic	137.92 (33.5)	113.59 (9.74)	173.41 (22.31)	<0.001
	Diastolic	88.78 (25.61)	69.97 (7.83)	116.22 (15.92)	<0.001
Urine protein level	0	1385 (58.02)	1385 (97.81)	0 (0.00)	<0.001
	1	29 (1.21)	29 (2.05)	0 (0.00)	
	2	339 (14.20)	0 (0.00)	339 (34.91)	
	3	363 (15.21)	0 (0.00)	363 (37.38)	
	4	271 (11.35)	2 (0.14)	269 (27.70)	
Ganda ethnicity	No, n (%)	992 (41.56)	612 (43.22)	380 (39.13)	0.05
	Yes, n (%)	1395 (58.44)	804 (56.78)	591 (60.87)	
Febrile illness	No, n (%)	1236 (71.86)	573 (75.69)	663 (68.85)	<0.01
	Yes, n (%)	484 (28.14)	184 (24.31)	300 (31.15)	

Pre-eclampsia was more severe in pregnancies delivered at earlier gestational ages (Fig. S4). For women with pre-eclampsia who delivered at 39 weeks’ gestation, mean systolic blood pressure is 168.95 (±19.14 SD), compared to 185.83 (±20.90 SD) at 28 weeks. Mean diastolic blood pressures at the same gestations in women without pre-eclampsia were 113.24 ± 14.53 and 122.58 ± 8.69 respectively. Higher proteinuria levels were also more common in women with pre-eclampsia who delivered at lower gestations.

Mean birth weight (without adjustment for gestational age) was significantly lower (p < 0.001) among pre-eclampsia cases (2.48 kg ± 0.81 SD) compared to normotensive pregnancies (3.06 kg ± 0.46 SD). Compared to international standards adjusted for sex and gestational age [13], birth weight was low overall within the study population: 17.30% of the birth weights in the normotensive group were ≤10th centile, compared to 32.54% of birth weights in the pre-eclampsia group (Fig. S8). By contrast, 6.25% of birth weights in the normotensive group were ≥90th centile, compared to 13.12% in the pre-eclampsia group (Fig. S8).

**Fig. 1:**
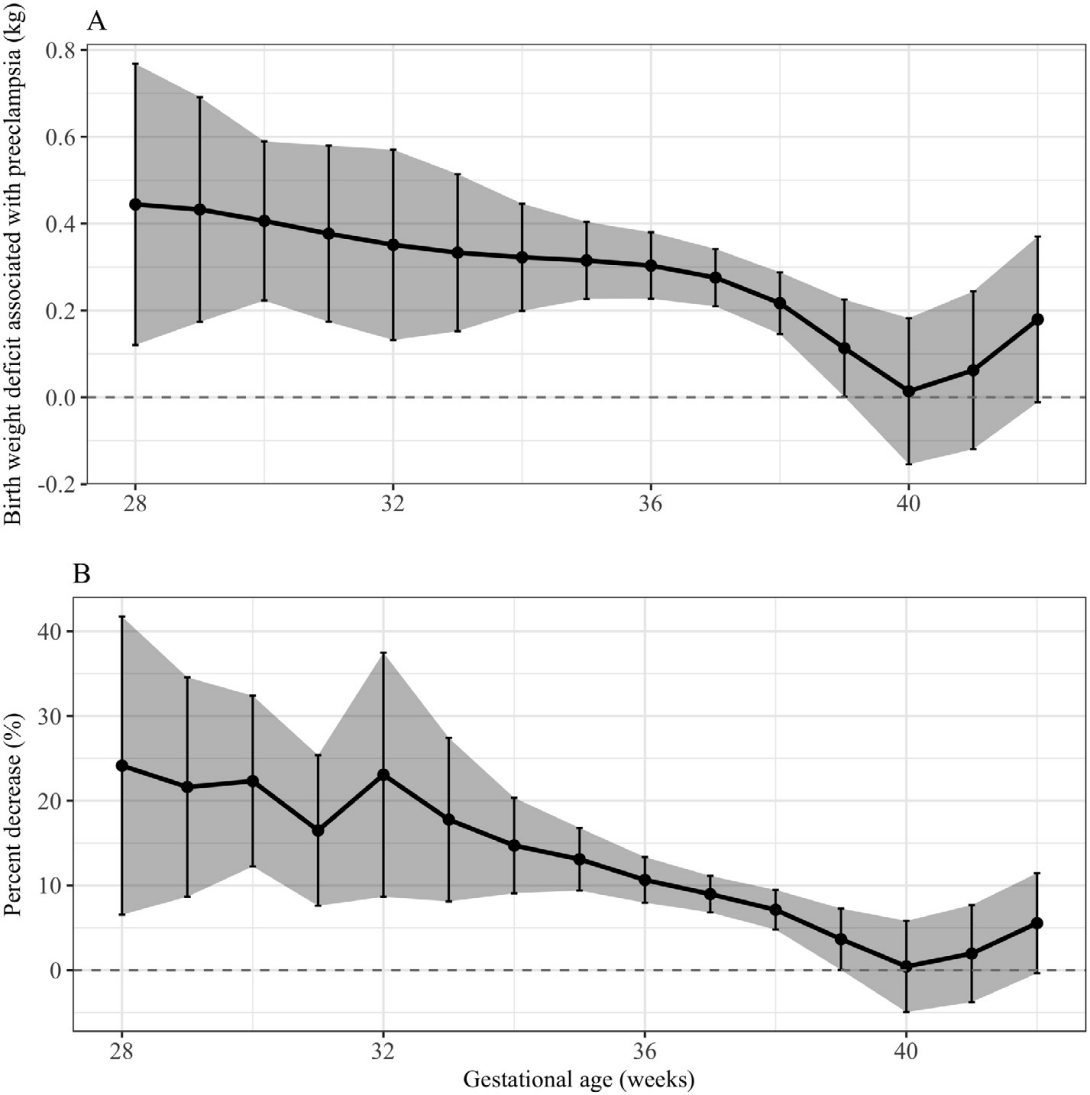
Mean birth weight difference between pre-eclampsia cases and normotensive pregnancies across gestational age at delivery. Solid black line represents the change in mean birth weight difference across gestation and grey shaded areas represent standard errors of the mean.

After controlling for maternal-fetal covariates in our spline model, the birth weight deficit associated with pre-eclampsia—that is, the model-adjusted difference in mean birth weight between the pre-eclampsia and normotensive groups—persisted throughout gestation. This birth weight deficit was greater at earlier gestational ages (p < 0.001; Fig. 1) and smaller at later gestational ages: 0.44 kg (±0.17 SE) for deliveries at 28 weeks, 0.33 kg (±0.02 SE) for those at 33 weeks, and 0.11 kg (±0.06 SE) at 39 weeks. There was a comparatively small widening in birth weight deficit for pregnancies delivered after week 40.

We investigated the possibility that the birth weight deficit in the pre-eclampsia group was higher at earlier gestational ages because women delivering earlier had more severe pre-eclampsia phenotypes. After propensity scoring, there was some attenuation of the gestational variation in the effect of pre-eclampsia, with a weaker impact of pre-eclampsia at earlier gestational ages. However, there are still clear gestational differences in the effect of pre-eclampsia on birth weight, even in the severity-matched analysis (Fig. S6). Thus we found only weak evidence to suggest that differences in disease severity across gestation can explain the gestational trend seen in Fig. 1. Even in this large sample of women who experienced pre-eclampsia (971 cases), the smaller numbers at lower gestational ages (Fig. S7) mean that estimate of the birth weight deficit at very low survivable gestational age is necessarily imprecise.

**Fig. 2:**
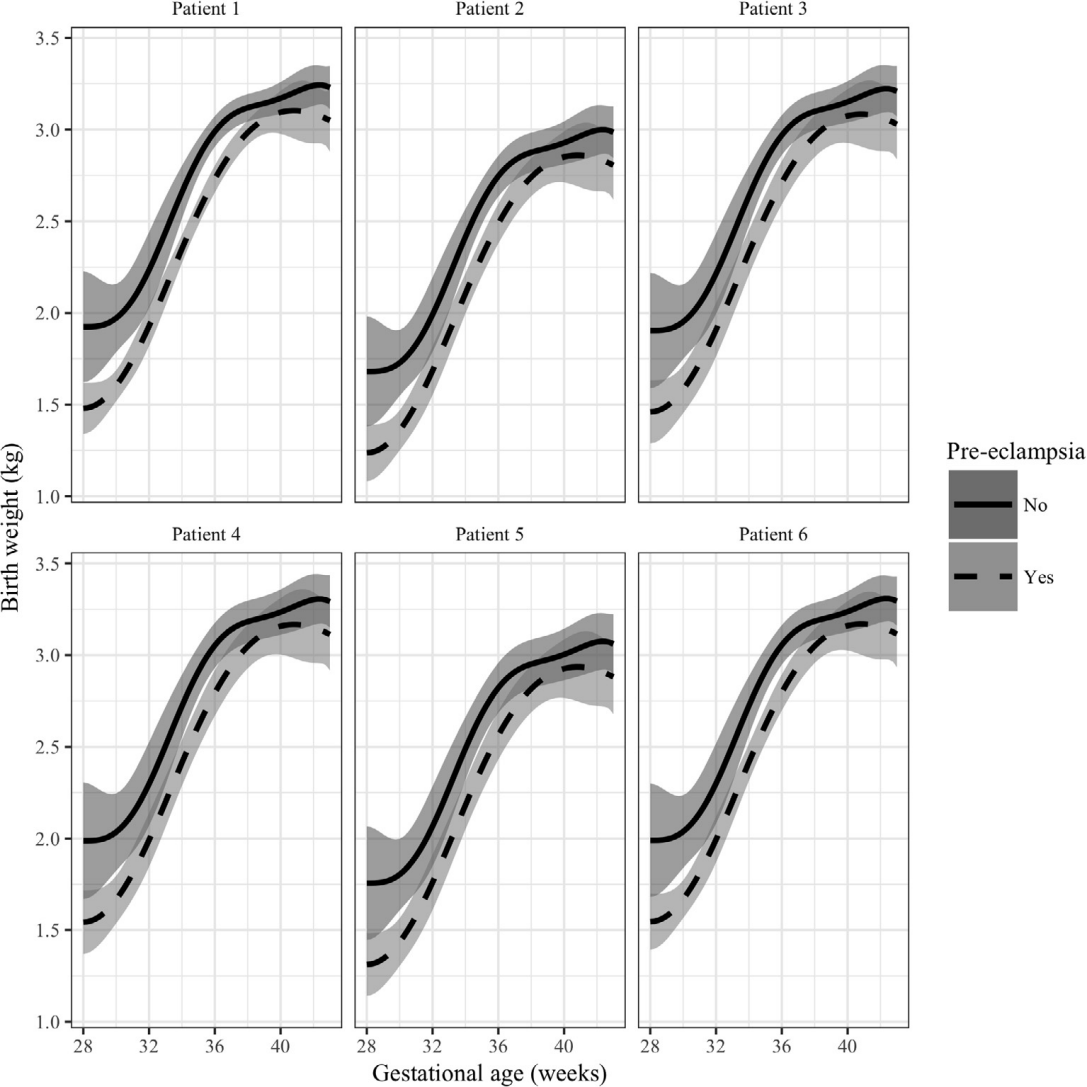
Predicted birth weight curves for six hypothetical patients. Each hypothetical patient has different combinations of the factors that have a statistically significant effect on birth weight. Predicted birth weight curves are shown for the same hypothetical patient with (dashed line) and without (solid line) pre-eclampsia. Grey shaded areas represent standard errors of the mean in both cases. Patient 1: 30 yr old, multiparous, HIV negative, mother in a skilled occupation with a live-born male infant. Patient 2: 20 yr old, multiparous, HIV negative, mother in a skilled occupation with a stillborn female infant. Patient 3: 15 yr old, primiparous, HIV positive, mother in a skilled occupation with a live-born male infant. Patient 4: 40 yr old, primiparous, HIV negative, mother in an unskilled occupation with a live-born female infant. Patient 5: 35 yr old, multiparous, HIV positive, mother in a professional occupation with a stillborn male infant. Patient 6: 40 yr old, multiparous, HIV negative, mother in a skilled occupation with a live-born male infant.

In our regression-spline model, factors other than pre-eclampsia significantly associated with lower birth weight included female sex of the baby (p < 0.001), stillbirth (p < 0.05), and having unskilled or no employment (p < 0.05). Factors significantly associated with higher birth weight included higher maternal age (p < 0.05) and primiparity (p < 0.001) (Table 2). To illustrate the magnitude of these effects, we created predicted birth weight curves for six hypothetical patients in the presence and absence of pre-eclampsia (Fig. 2). While there is a distinct separation in the curves with respect to pre-eclampsia in all hypothetical patients, the similarity in curves between patients indicates that other maternal-fetal covariates do not have practically significant effects on birthweight across gestational age, despite being statistically significant.

**Table 2:** Variation in birth weight attributable to maternal and fetal factors. The percentage of total variability in birth weight ascribed to each factor is quantified using the partial R-squared value from the primary outcome model in each of the three gestational age categories (<34 weeks, 34–36 weeks, and > 36 weeks).

Factor	Percentage of birth weight variability (%)		
	<34 weeks	34–36 weeks	>36 weeks
Gestational age	84.66	82.65	89.86
Pre-eclampsia	7.11	10.53	7.79
Other known maternal-fetal characteristics	0.73	0.16	0.06
Unexplained variation	7.50	6.66	2.29

Finally, our analysis of variance showed that at low gestational ages (<34 weeks), pre-eclampsia predicts 7.1% of the variation in birth weight, versus 0.7% for all other maternal-fetal characteristics except gestational age (Table 2). At higher gestational ages (>37 weeks), pre-eclampsia predicts 7.8% of the variation in birth weight, versus 0.1% for all other characteristics except gestational age. Gestational age itself accounts for more than 85% of the variation in birth weight (Fig. S3).

## 4 Discussion

We show that, in urban Uganda, maternal pre-eclampsia is the dominant influence on birth weight across all gestations. In our population, pre-eclampsia alone accounts for approximately ten times more of the variability in birth weight than all other identified risk-factors combined. We demonstrate that although the effect of pre-eclampsia on birth weight is consistently present, there is substantial reduction in this birth weight deficit at later gestational ages.

Although it is clear that mothers with more severe pre-eclampsia tend to deliver at earlier gestations, the higher birth weight deficit at earlier gestations still persists even after controlling for pre-eclampsia severity using matching. The evidence is thus insufficient to conclude definitively that increased growth restriction in pregnancies delivered at lower gestational ages is explained by higher pre-eclampsia severity. Pre-eclampsia severity is defined clinically in terms of maternal symptoms, but may also be considered in terms of direct placental impact. It is important to note that pregnancies delivered earlier may have more adverse placental phenotypes, and this may be better correlated to fetal growth restriction than maternal symptoms. This is an important consideration for future research.

The particular setting of our study, in a difficult-to-study maternity population, is a major strength and may be relevant to the observed dominance of pre-eclampsia as a risk factor for fetal growth restriction. The urban Ugandan population is relatively treatment-naïve in the context of pre-eclampsia, due to the high prevalence of late presentation. This provides a rare opportunity to study the natural history of pre-eclampsia, which is often masked by early detection, medical treatment, and careful timing of delivery, yet is evident in this unusual data set. Furthermore, the high prevalence of pre-eclampsia in this setting and contemporaneous data collection strategy has enabled the collection of a large number of cases of pre-eclampsia of high severity. The sophisticated and flexible modelling employed in our study has enabled us to quantify the magnitude of risk associated with pre-eclampsia and other observable characteristics.

Our analysis is subject to several limitations. First, there are several degrees of freedom in our process: specification of the spline basis, variable selection, and form of the interaction between gestational age and pre-eclampsia. The need for a choice regarding each of these is inherent in any parametric model specification, and we have tried to minimize researcher degrees of freedom here by using stepwise selection to decide the form of the model (see Appendix S6). Second, our ability to explore in more detail the relationship between pre-eclampsia severity and birth weight was somewhat limited by smaller sample sizes at low gestational ages (see Appendix S5).

Our finding that pre-eclampsia severity is not a good correlate of the magnitude of fetal growth restriction is in keeping with the findings of smaller studies of severe pre-eclampsia in higher income settings [20]. Our results suggest that the timing of delivery is a better correlate of fetal growth restriction than maximal disease severity. There are at least two possible explanations for this: the first is that the maximal severity in early onset cases is never reached because the disease process is attenuated by delivery. Had these pregnancies continued to later gestations, they may have manifested a more severe phenotype than the later-delivered pregnancies with which they were propensity matched in our analysis. The second possibility is that pregnancies where the maternal symptoms of pre-eclampsia manifest earlier have a more severe fetal phenotype than pregnancies with equal pre-eclampsia severity but later manifestation of maternal symptoms. Recognition of pre-eclampsia in urban Uganda and other LM HDI settings relies on maternal symptoms prompting attendance for obstetric care. Thus our results may reflect a correlation between the timing of maternal symptom emergence and the severity of fetal growth restriction.

Although an association between pre-eclampsia and fetal growth restriction is described in other contexts, the magnitude of the growth restriction ascribed to pre-eclampsia is rarely as large as in this cohort [10], [21]. In our population, premature babies of mothers who experienced pre-eclampsia were up to 25% (0.58 kg) smaller than their normotensive counterparts. This is a clinically significant finding, particularly in a setting where survival to hospital discharge at 28–30 weeks’ gestation is by no means assured [22]. The elevated mortality risk for these babies persists even after leaving hospital, as a discharge weight of ≤1500 g in urban Uganda is associated with a 20% risk of death within three months [23]. We also found a significantly greater proportion of LGA (≥90^th^ centile) babies in the pre-eclampsia group than in the normotensive group. This finding is an important subject for future research, as it may be independently associated with other adverse perinatal outcomes.

In the sub-Saharan African clinical setting, simple heuristics to guide practice are often invaluable. The ability to recognize babies at highest risk of being born at low birth weight maximizes the chance of appropriate interventions. Understanding which factors are the key determinants of birth weight in a particular context also allows the formulation of strategies targeted at reducing the incidence of low birth weight and hence improving perinatal survival. Our results suggest that perinatologists should regard the timing of delivery in the context of pre-eclampsia as a better predictor for associated fetal growth restriction than indices of pre-eclampsia severity.

## Funding

CA is supported by an Isaac Newton Trust[12.21(a)]/Wellcome Trust ISSF [105602/Z/14/Z]/ University of Cambridge Joint Research Grant. This work was funded by the Wellcome Trust (094073/Z/10/B), and a Wellcome Trust Uganda Postdoctoral Fellowship in Infection and Immunity held by AN, funded by a Wellcome Trust Strategic Award, grant number 084344. Supported by NURTURE fellowship to AN, grant number D43TW010132. This work was also supported through the DELTAS Africa Initiative (grant number 107743/Z/15/Z). The DELTAS Africa Initiative is an independent funding scheme of the African Academy of Sciences (AAS)’s Alliance for Accelerating Excellence in Science in Africa (AESA) and supported by the New Partnership for Africa’s Development Planning and Coordinating Agency (NEPAD Agency) with funding from the Wellcome Trust (grant number 107743/Z/15/Z) and the UK government. The views expressed in this publication are those of the author(s) and not necessarily those of AAS, NEPAD Agency, Wellcome Trust or the UK government. JES acknowledges the support of a T32 fellowship from the U.S. National Institutes of Health.

## CRediT authorship contribution statement

**Annettee Nakimuli:** Data curation, Funding acquisition, Investigation, Methodology, Project administration, Resources, Writing - original draft. **Jennifer E. Starling:** Data curation, Formal analysis, Investigation, Methodology, Writing - review & editing. **Sarah Nakubulwa**, **Imelda Namagembe**, **Musa Sekikubo**, **Eve Nakabembe:** Investigation, Methodology, Writing - review & editing. **James G. Scott:** Conceptualization, Formal analysis, Methodology, Supervision, Writing - review & editing. **Ashley Moffett:** Conceptualization, Supervision, Writing - review & editing. **Catherine E Aiken:** Conceptualization, Data curation, Formal analysis, Writing - original draft.
