# Supplementary material for: Relative impact of pre-eclampsia on birth weight in a low resource setting: A prospective cohort study
Source: Pregnancy Hypertens. 2020 Jul;21:1–6. doi: 10.1016/j.preghy.2020.04.002 (PMC7450268; doi:10.1016/j.preghy.2020.04.002)
Supplement: Supplementary data 1 [file mmc1.docx]

Impact of pre-eclampsia on birth weight in sub-Saharan Africa: a prospective cohort study

Annettee Nakimuli^1^, Jennifer E. Starling^2^, Sarah Nakubulwa^1^, Imelda Namagembe^1^, Musa Sekikubo^1^, Eve Nakabembe^1^, James G. Scott^2^, Ashley Moffett^3^, Catherine E. Aiken^4*^

^1^ Department of Obstetrics and Gynaecology, Makerere University and Mulago National Referral Hospital, Kampala, Uganda.

^2^ Red McCombs School of Business and Department of Statistics and Data Sciences, University of Texas at Austin, Texas, USA

^3^ Department of Pathology and Centre for Trophoblast Research, University of Cambridge, Cambridge, United Kingdom

^4^ Department of Obstetrics and Gynaecology, University of Cambridge, Box 223, The Rosie Hospital and NIHR Cambridge Biomedical Research Centre, Cambridge CB2 0SW, United Kingdom

*Corresponding author:

Telephone: +44(0)1223 336871 Email: [cema2@cam.ac.uk](mailto:cema2@cam.ac.uk)

Running title:

Influence of pre-eclampsia on birth weight in urban Uganda

Abstract:

This appendix has been provided by the authors to give readers additional information about their work. It provides detail for all model selection processes and statistical analyses of birth-weight versus maternal pre-eclampsia status across gestation.

# Appendix S1. Malaria exposure during pregnancy

One of the maternal-fetal characteristics available was self-reported febrile illness during pregnancy, as a proxy for malaria during pregnancy. Presence or absence of febrile illness was known for 1,720 of the 2,387 subjects (72.1%). An important preliminary step in specifying a model for birth-weight versus pre-eclampsia was to determine whether febrile illness is a significant predictor of birth-weight for these 1,720 women where data is available.

To assess significance of febrile illness as a predictor of birth-weight, we fit a regression spline model on the subset of observations where febrile illness was reported. Our spline model includes a cubic I-spline basis for gestational age with 7 degrees of freedom. Our model adjusts for maternal age, parity, maternal job type, ethnicity, HIV status, whether the infant was stillborn, infant sex, and maternal pre-eclampsia status. Our model also includes two-way interactions between basis elements and pre-eclampsia status, and infant sex, respectively; as well as the three-way interactions between pre-eclampsia status, infant sex, and spline basis functions. We assessed the significance of the model coefficient for febrile illness using a t-test. This analysis suggested that self-reported febrile illness was not significantly associated with birth weight, with p-value 0.607. Febrile illness was therefore excluded during the stepwise variable selection process, and further model selection was performed using all available data.

# Appendix S2. Methods for stepwise model selection

Our primary analysis assessed the relationship between pre-eclampsia and birth weight, using regression splines to fit a smooth nonlinear model for birth weight as a function of both gestational age and pre-eclampsia status, using cubic splines. The model was chosen via a three-step selection process. First, we chose the number of basis coefficients, to determine number and location of spline knots. Second, we performed variable selection to determine which other maternal-fetal covariates should be included in the model. Finally, we specified the form of the interaction between pre-eclampsia and gestational age.

We begin by choosing the number of basis coefficients (i.e. spline knots), following a previously published procedure ^1^. Each model’s Akaike inference criterion (AIC) was evaluated across a grid of candidate values for the number of knots. For each possible model, knots were placed at regularly spaced quantiles of the birth weight distribution. The model with the minimum AIC value was chosen. At this initial knot-selection step, we included all other maternal-fetal covariates: maternal age, parity, maternal job type, Ganda ethnicity, HIV status, and infant sex. We also included two-way interactions between pre-eclampsia and gestational age (i.e. the spline basis functions), and two-way interactions between basis elements and infant sex. We also included a three-way interaction between gestational age, infant sex, and pre-eclampsia. This model allows for potential differences in fetal growth over a pregnancy for (i) male versus female infants, (ii) infants of mothers with pre-eclampsia, and (iii) differences in the impact of pre-eclampsia on the growth of male versus female infants.

Candidate degrees of freedom evaluated ranged from two to seven. After initial selection on the full model, we repeated this process for a selection of subsets of the full model, in order to assess sensitivity of the optimal degrees of freedom to the covariates included in the model. We removed the three-way interactions, then the two-way and three-way interactions, and then various main effects, one at a time. For each nested model, we obtained the optimal degrees of freedom. We then assessed the range of optimal degrees of freedom selected over all models, and chose the degrees falling in the middle of this range, to balance regularization and allowing for sufficient model complexity. To ensure results were not specific to choice of information criterion, we repeated the process using the Bayesian information criterion (BIC) in place of AIC.

We then perform stepwise selection to determine which other maternal-fetal covariates are retained in the model, followed by a likelihood ratio test to determine the optimal form of the interaction between gestational age and pre-eclampsia. Candidate interaction forms are: (i) no interaction, (ii) a linear interaction (so that pre-eclampsia may shift birth-weight up or down uniformly across all gestational ages, but not differently at different gestational ages), and (iii) an interaction between the gestational age spline basis elements and pre-eclampsia (allowing pre-eclampsia to modify the shape of the curve relating birthweight to gestational age).

The result of this model selection process is used in our primary outcome analysis.

**Appendix S3. Methods for primary outcome analysis**

We assessed the mean effect of pre-eclampsia on birth-weight across gestational age using the model selected in Appendix 2. The mean effect of pre-eclampsia across gestational age is calculated as the difference in estimated mean birth-weight for women with pre-eclampsia versus without, at each gestational age. Additionally we aim to quantify the magnitude of the effect of pre-eclampsia on birth-weight in comparison to other maternal-fetal covariates included in our model.

We begin by generating two synthetic model predictions for each observation in the dataset, regardless of actual pre-eclampsia status: one predicted birth-weight where that observation has pre-eclampsia set to *no*, and another where pre-eclampsia is set to *yes*. We then calculated the difference in estimated birth-weight in presence versus absence of pre-eclampsia for each prediction. We use these predicted differences to calculate the mean differences and their standard errors at each gestational age. This is the equivalent of Friedman’s two-dimensional partial dependence function of birthweight on gestational age and pre-eclampsia ^2^.

We use standard results on linear models to calculate standard errors for the mean differences in birth-weight for pre-eclampsia versus non-pre-eclampsia cases at each gestational age. Let $\overset{̂}{y}(a,1,x)$ represent the estimated mean birth weight for gestational age $a$, pre-eclampsia, and covariates $x$. Similary, let $\overset{̂}{y}(a,0,x)$ be the corresponding estimated mean birth weight for the same gestational age and covariates, without pre-eclampsia. The mean difference at gestational age *a* is$\overset{̂}{y}(a,1,x)-\overset{̂}{y}(a,0,x)$, and we are interested in its standard error, $\mathrm{SE}\left( \overset{̂}{y}(a,1,x)-\overset{̂}{y}(a,0,x) \right)$. Since the only interaction in the model is between gestational age and pre-eclampsia, this distance is the same for all $x$ configurations. We can therefore write the mean difference as $\overset{̂}{r}(a,1)-\overset{̂}{r}(a,0)$, where

$$\overset{̂}{r}(a,j)={X_{rj}}^{T}{\overset{̂}{\beta}}_{r}$$

and $X_{rj}$ is the design matrix for the model with pre-eclampsia set to $j\in\left\{ 1,0 \right\}$ for all observations, and including only the columns related to pre-eclampsia and gestational age; columns for covariates $x$ are excluded. Then ${\overset{̂}{\beta}}_{r}$ is the vector of estimated coefficients related to pre-eclampsia and gestational age.

We can then calculate the standard error at each gestational age using standard variance formulas:

$$\mathrm{Var}\left[ \overset{̂}{r}\left( a,1 \right)-\overset{̂}{r}\left( a,0 \right) \right]$$

$$=Var\left[ \overset{̂}{r}\left( a,1 \right) \right]+Var\left[ \overset{̂}{r}\left( a,0 \right) \right]-2Cov\left[ \overset{̂}{r}\left( a,1 \right),\overset{̂}{r}\left( a,0 \right) \right]$$

$$=Var[{X_{r1}}^{T}{\overset{̂}{\beta}}_{r}]+Var[{X_{r0}}^{T}{\overset{̂}{\beta}}_{r}]+X_{r1}Var[{\overset{̂}{\beta}}_{r}]{X_{r0}}^{T}$$

Note that we can easily obtain these variances from the linear model output in R, as follows. Let $X_{r}$ represent a stacked model matrix, excluding columns related to covariates $x$, for a single patient, such that $X_{r}=[X_{r1}^{T}|X_{r0}^{T}]^{T}$. We can then calculate

$$\Sigma=X_{r}Cov({\overset{̂}{\beta}}_{r})X_{r}^{T}$$

resulting in $\Sigma$ as a matrix with four quadrants: comparing pre-eclampsia to itself in the top left, comparing non-pre-eclampsia to itself on the bottom right, and then comparing pre-eclampsia to non-pre-eclampsia on the off-diagonals. We obtain the correct variances at each gestational age by taking the diagonals of each of these blocks; the top left and bottom right diagonal entries are $\mathrm{Var}[\overset{̂}{r}(a,1)]$ and $\mathrm{Var}[\overset{̂}{r}(a,0)]$, respectively, and the off-diagonal blocks’ diagonals, which are of course identical, each representing $\mathrm{Cov}[\overset{̂}{r}(a,1),\overset{̂}{r}(a,0)]$.

We then conducted an analysis of variance as a way to quantify the percent of variation explained by gestational age, pre-eclampsia, other maternal-fetal characteristics, and residual variation, within three gestational age groups: $<34$ weeks, $34-36$ weeks, and $>36$ weeks. In data sets like ours, where the predictors are correlated, the percent-variation explained in an ANOVA depends on the order in which predictors are added to the model. Thus to assess the relative contributions of the variables, we added all gestational age spline terms first and computed the model’s R^2^, both overall and separately by gestational age category. We then added all maternal-fetal control variables except for pre-eclampsia and computed the improvement in R^2^, again both overall and separately by gestational age category. Finally, we added pre-eclampsia status and once again computed the improvement in R^2^. By adding pre-eclampsia status last, our calculation of the percent-variation explained by pre-eclampsia is adjusting for the variation in birthweight that is explained both by gestational age and by all other predictors in the model.

**Appendix S4. Methods for secondary outcome analysis**

Our secondary analysis focuses on the severity of pre-eclampsia. Recall that our primary analysis showed more severe growth restriction for pre-eclampsia cases at earlier gestational ages. Our secondary analysis aimed to determine whether these gestational differences in growth restriction are explained by the tendency of pre-eclampsia cases to be more severe at earlier gestational ages. We measure severity using the three outcome markers described in the Methods section of the paper: (i) maximum systolic blood pressure measured during delivery, (ii) maximum diastolic blood pressure measured during delivery, and (iii) maximum proteinuria level on dipstick. All markers indicate a tendency for pre-eclampsia cases to be more severe in pregnancies delivered at earlier gestational ages (Figure S4).

We then used propensity score matching to construct a severity-matched data set across gestational ages—that is, a subset of the data for which there are no systematic differences in pre-eclampsia severity across gestational age. This severity-matched dataset was constructed by first retaining all pregnancies without pre-eclampsia. Then for cases with pre-eclampsia, we performed propensity score matching to pair cases of pre-eclampsia at high gestational ages (>36 weeks) to those with similar severity at low ($\leq$ 36 weeks) gestational ages. Pre-eclampsia cases without a match were discarded. By retaining only these matched pre-eclampsia pairs, we remove observations where there is no observation of similar severity at the opposite end of the gestational age spectrum. The result is a data set where the markers of pre-eclampsia severity are similar, on average, across all gestational ages.

To estimate propensity scores, we constructed a random forest classification model using proteinuria level and maximum systolic and diastolic blood pressure as features for predicting whether gestational age is below 36 weeks. For a discussion of the benefits of a random forest model in propensity-score modeling, we refer interested readers to Lohr et al. (2018)^3^.

We then re-fit our regression-spline model to the matched dataset and re-calculated the mean differences in birth weight for pre-eclampsia cases versus controls at each gestational age, to assess whether this rebalancing process attenuated the gestational differences in the effect of pre-eclampsia on birthweight.

The final output of our secondary analysis is a modified growth-restriction curve for pre-eclampsia across gestational age, which can be compared to the growth restriction curve estimated on the full data set (Figure 1 in main paper). Under the hypothesis that the gestational differences in growth restriction can be fully explained by the presence of more severe cases occurring at earlier gestational ages, we expect to see a flatter overall curve on the matched data set, i.e. one where growth restriction is roughly constant across gestational ages for pre-eclampsia cases versus controls.

**Appendix S5. Results and interpretation**

The first step of the model selection process – choosing the degrees of freedom for the regression splines model – resulted in the choice of five degrees of freedom. Optimal values generated by the various models ranged from four to six; see Figure S2. The range of optimal internal knot values across all models was 1–3, or 3–6 degrees of freedom. We selected 5 degrees of freedom, or 2 internal knots, to balance model regularization with quality of fit (Figure S2). We found that results were not specific to choice of information criterion; using BIC in place of AIC resulted in an identical range of optimal degrees of freedom values.

The second step of the model selection process – variable selection for other maternal-fetal characteristics – resulted in ethnicity being excluded from the model. The process also excludes the two-way interaction between gestational age and infant sex, as well as the three-way interaction between gestational age, infant sex, and pre-eclampsia; small sample sizes at lower gestational ages make it unlikely that there is sufficient statistical power to detect potential three-way interactions. The following maternal-fetal characteristics are included in the model: stillbirth, maternal age, infant sex, parity, HIV status, and maternal job type.

The third step of the model selection process – choosing the form of the interaction between gestational age and pre-eclampsia – resulted in including the interaction between gestational age basis elements and pre-eclampsia (p-value <0.001). This result is consistent with previous steps in the model selection process. Where any time variable selection was performed this interaction was included in the resulting model.

In summary, the model selected for analyzing the primary outcome models birth weight dependent on a cubic spline basis for gestational age with five degrees of freedom. The model includes the interaction between pre-eclampsia and the basis elements, as well as main effects for pre-eclampsia status, stillbirth, maternal age, infant sex, parity, HIV status, and maternal job type.

Figure 1 and the Results section of the paper discuss results for modeling the mean difference in birth-weight for women with pre-eclampsia versus not, across gestational ages. In addition, Table A1 gives detail on mean differences and their standard errors at each gestational age. We also include Figure S3 as a visual representation of the percent of variation attribution findings discussed in Results.

In the secondary analysis, Figure S5 illustrates successful propensity score matching. Figure S6 illustrates the mean differences and standard errors across gestational age for both the original and propensity score-matched datasets. Evidence was insufficient to conclude that increased growth restriction is driven by higher severity pre-eclampsia in pregnancies delivered at lower gestational ages. We also note that small sample sizes at early gestational ages likely limit power to detect any potential attenuation (Figure S7). We have also contextualized the birth weights in our cohort overall, by plotting the birth weight centiles of both the pre-eclampsia and normotensive groups according to international Intergrowth 21^st^ standards ^4^ (Figure S8).

# Appendix S6. Discussion

We elaborate on limitations of our analysis discussed in the main paper. We note two additional limitations here. We expect the estimated growth curves to be monotone non-decreasing. Our model is fit using I-splines, which imposes non-decreasing monotonicity for each spline segment; we see that this results in growth curves that look as expected in Figure 1. However, imposing a stricter monotonicity condition in a nonparametric functional regression setting would address the limitations discussed in the paper regarding model selection and assumption of basis selection and form of interactions. This is an area of future work.

The second limitation is potential effect of rounding bias in birth-weight. Birth weights are often rounded to the nearest 100 grams, due to service demands at the time of data collection at the Mulago National Referral Hospital. A limitation of our analysis is that we do not adjust for this potential heaping bias. We do note that 53% of the dataset, or 1279 observations, have birth weights recorded to the nearest 100 grams. Some of these may be measured precisely, while others may be rounded. Another area of future work is to design a model incorporating this uncertainty about the true birth-weights for the cases potentially rounded to the nearest hundred grams.

The potential rounding bias in birth-weight motivated the decision to perform regression on the mean, as opposed to performing a logistic regression where the response was being below a specific birth weight threshold (such as the lowest decile of the Intergrowth 21st reference charts ^4^). Regression for the mean is unbiased in the presence of rounding, whereas regression for tails tends to suffer from bias in this case.

References

1 Likhachev, D. V. Selecting the right number of knots for B-spline parameterization of the dielectric functions in spectroscopic ellipsometry data analysis. *Thin Solid Films* **636**, 519-526, doi:10.1016/j.tsf.2017.06.056 (2017).

2 Friedman, J. Greedy Function Approximation: A Gradient Boosting Machine. *Annals of Statistics* **29**, 1189-1232 (2001).

3 Lohr, P. A., Starling, J. E., Scott, J. G. & Aiken, A. R. A. Simultaneous Compared With Interval Medical Abortion Regimens Where Home Use Is Restricted. *Obstet Gynecol* **131**, 635-641, doi:10.1097/AOG.0000000000002536 (2018).

4 Villar, J. *et al.* International standards for newborn weight, length, and head circumference by gestational age and sex: the Newborn Cross-Sectional Study of the INTERGROWTH-21st Project. *Lancet* **384**, 857-868, doi:10.1016/S0140-6736(14)60932-6 (2014).

# Appendix figures legends

Figure S1: Cohort diagram

Figure S2: Optimal degrees of freedom for models of varying complexity. Solid black lines represent the AIC for each model, each fit at every candidate degrees of freedom on the x-axis. The grey shaded area represents the range of optimal degrees of freedom values identified by any of the models, ranging from four to six degrees.

Figure S3: Percent of variation attributable to each category of effects, at low, medium, and high gestational ages: gestational age, other maternal-fetal characteristics, pre-eclampsia, and residual variation. The impact of pre-eclampsia dominates the smaller impact of other maternal-fetal characteristics in all gestational age groups. As expected, gestational age is the predominant source of variation in all groups.

Figure S4: Severity of pre-eclampsia across gestational age, as indicated by three pre-eclampsia markers for deliveries across gestational age: (A) maximum systolic blood pressure measured during the delivery episode, (B) maximum diastolic blood pressure measured during the delivery episode, and (C) maximum proteinuria level on dipstick. All markers indicate tendency for pre-eclampsia cases to be more severe at deliveries occurring at earlier gestational ages. Shaded grey areas represent standard errors of the mean.

Figure S5: Results of propensity score matching. The top row represents the original dataset, and the bottom row represents the propensity-score-matched dataset. In all panels, dark grey represents cases with pre-eclampsia, and light grey represents cases without pre-eclampsia.

Figure S6: Mean birth-weight differences for cases with versus without pre-eclampsia versus, and their standard errors, across gestational age – similar to Figure 1. Red represents the original dataset (replicating Figure 1), while blue represents the propensity score-matched dataset.

Figure S7: Sample sizes across gestational age for pregnancies where pre-eclampsia is present versus absent. Top panel illustrates sample sizes for female infants; bottom panel gives sample sizes for male infants. Sample sizes are small at early gestational ages for both infant sexes, both with and without pre-eclampsia.

Figure S8: Cohort birth weight centiles according to Intergrowth 21^st^ reference standards
