# Supplementary material for: Relative impact of pre-eclampsia on birth weight in a low resource setting: A prospective cohort study
Source: Pregnancy Hypertens. 2020 Jul;21:1–6. doi: 10.1016/j.preghy.2020.04.002 (PMC7450268; doi:10.1016/j.preghy.2020.04.002)
Supplement: Supplementary data 2 [file mmc2.pdf]

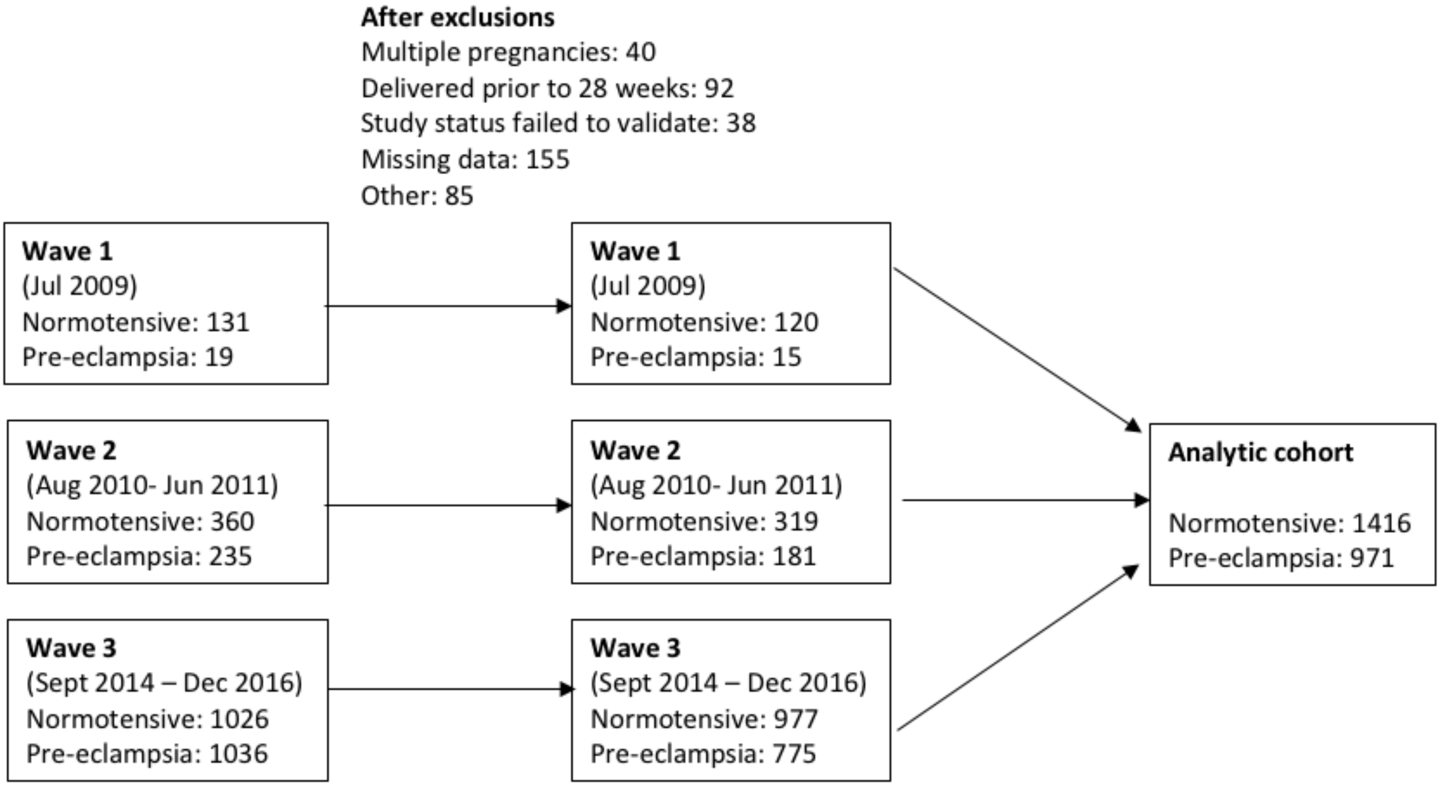
After exclusions
Multiple pregnancies: 40
Delivered prior to 28 weeks: 92
Study status failed to validate: 38
Missing data: 155
Other: 85
Wave 1
(Jul 2009)
Normotensive: 131
Pre-eclampsia: 19
Wave 2
(Aug 2010- Jun 2011)
Normotensive: 360
Pre-eclampsia: 235
Wave 3
(Sept 2014 – Dec 2016)
Normotensive: 1026
Pre-eclampsia: 1036
Wave 1
(Jul 2009)
Normotensive: 120
Pre-eclampsia: 15
Wave 2
(Aug 2010- Jun 2011)
Normotensive: 319
Pre-eclampsia: 181
Wave 3
(Sept 2014 – Dec 2016)
Normotensive: 977
Pre-eclampsia: 775
Analytic cohort
Normotensive: 1416
Pre-eclampsia: 971
