# Supplementary figures and images for: Relative impact of pre-eclampsia on birth weight in a low resource setting: A prospective cohort study
Source: Pregnancy Hypertens. 2020 Jul;21:1–6. doi: 10.1016/j.preghy.2020.04.002 (PMC7450268; doi:10.1016/j.preghy.2020.04.002)

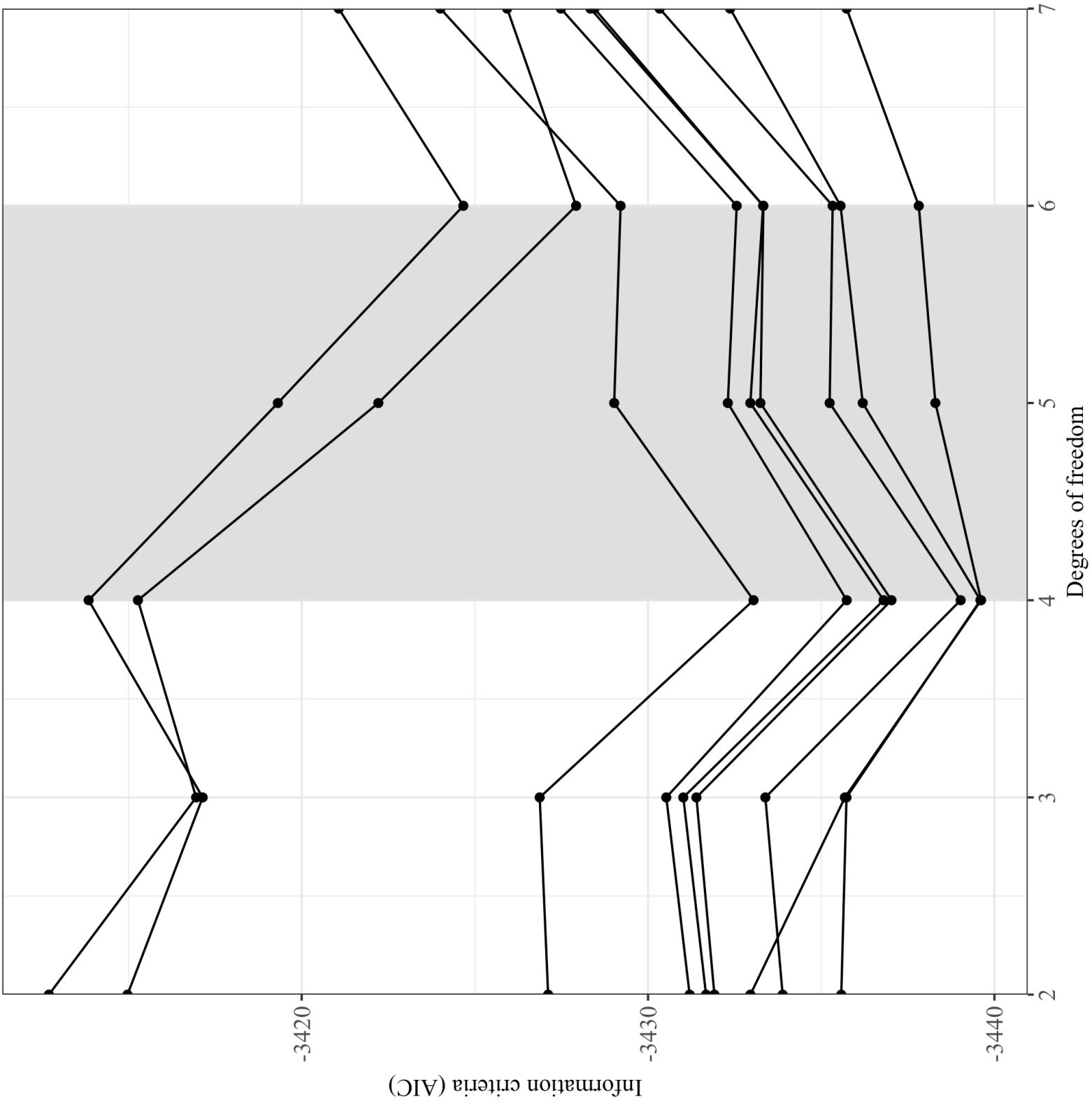

Supplement: Supplementary data 3 [file mmc3.pdf]

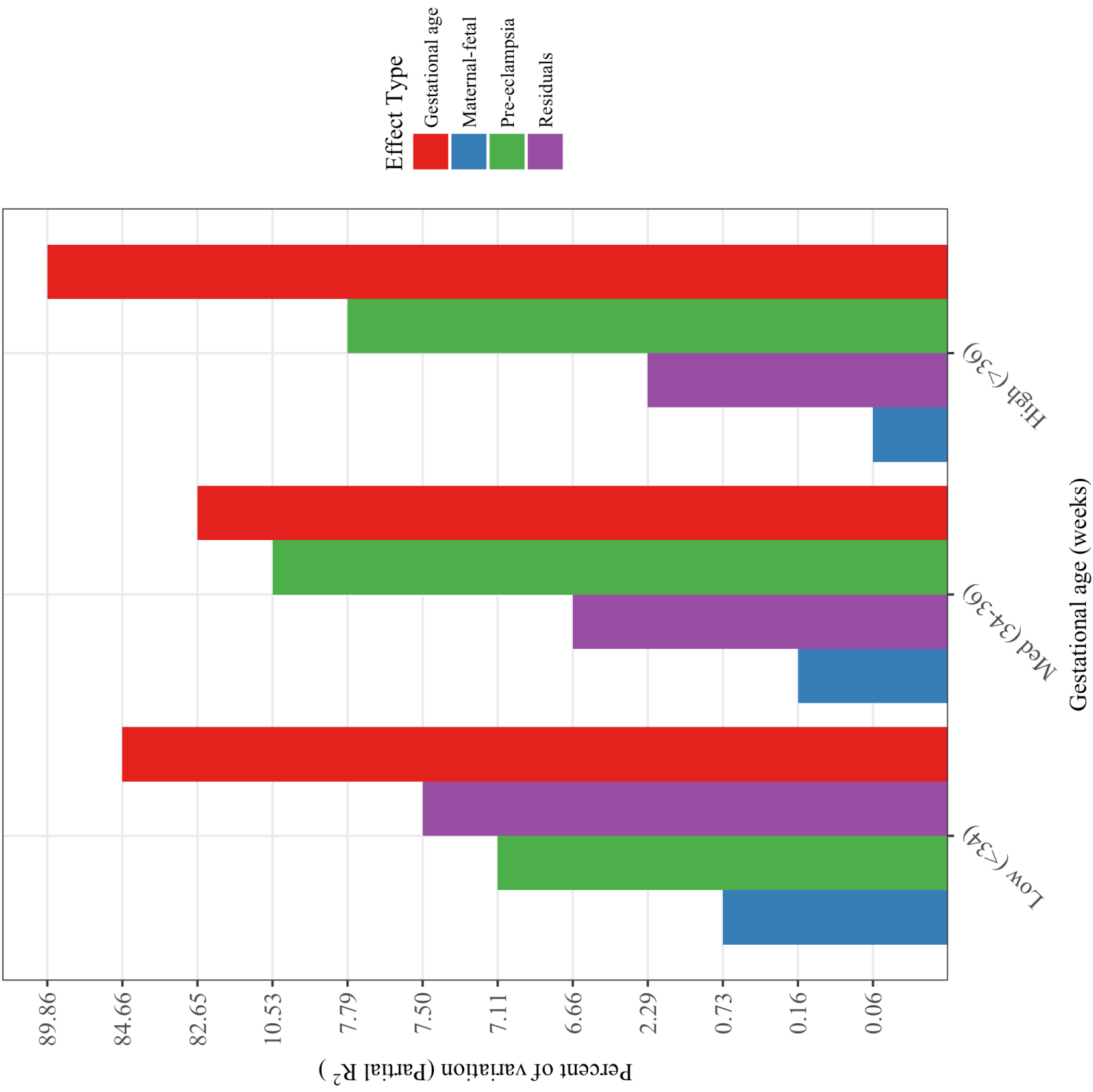

Supplement: Supplementary data 4 [file mmc4.pdf]

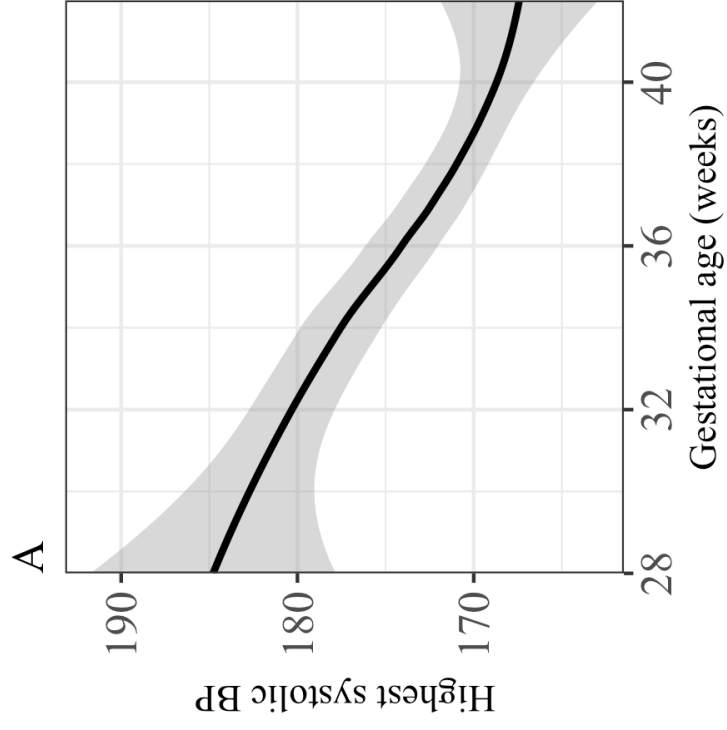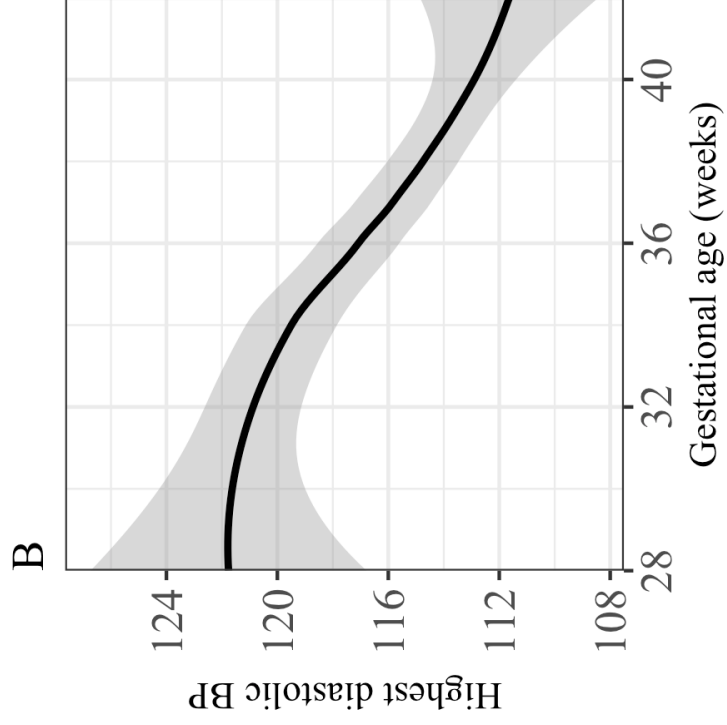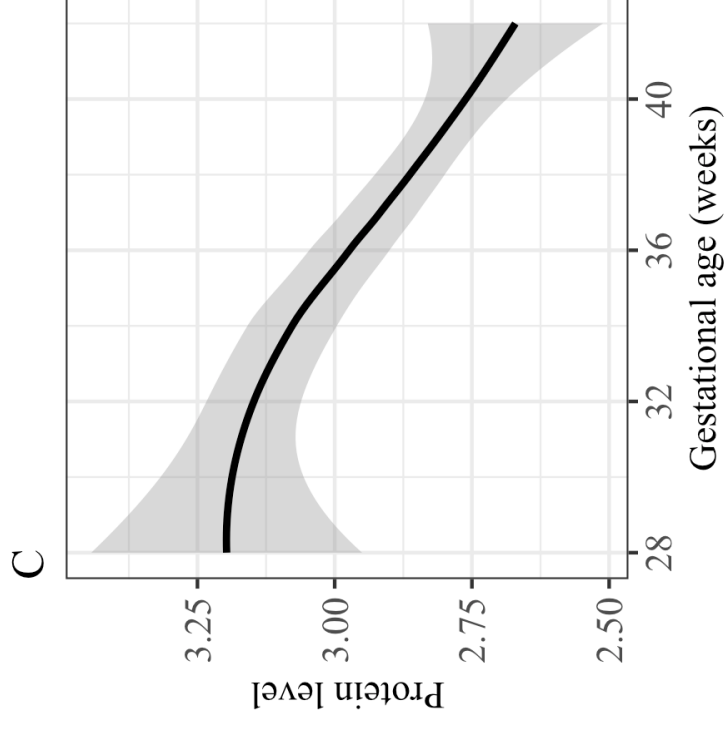

Supplement: Supplementary data 5 [file mmc5.pdf]

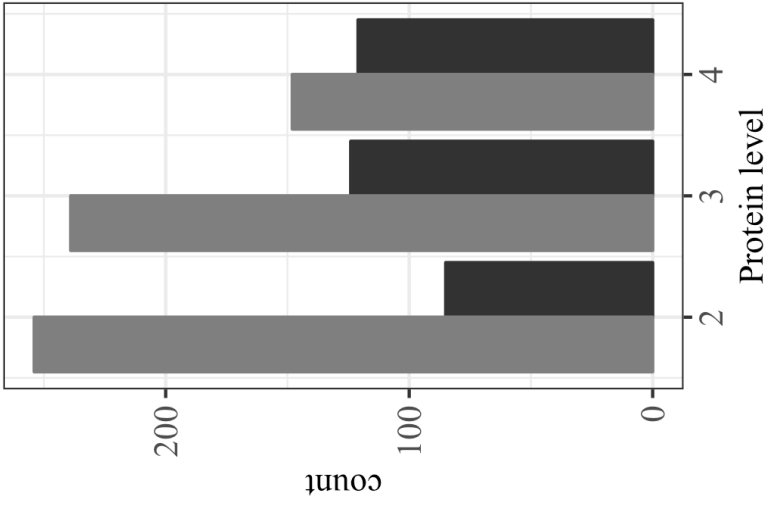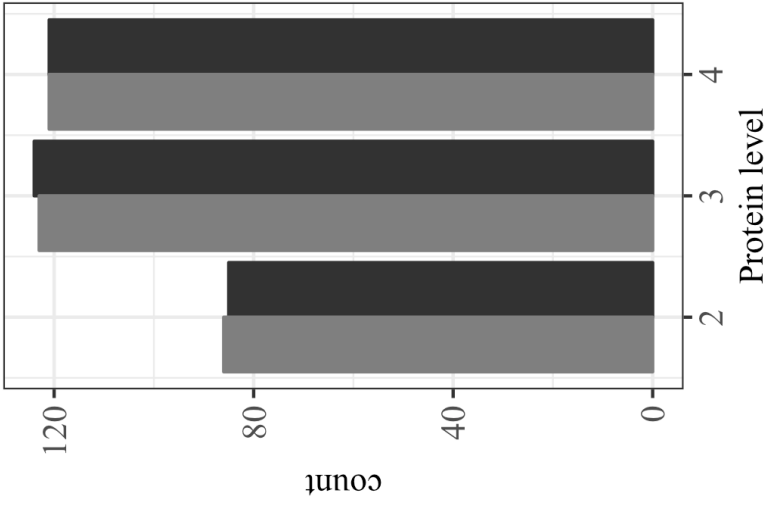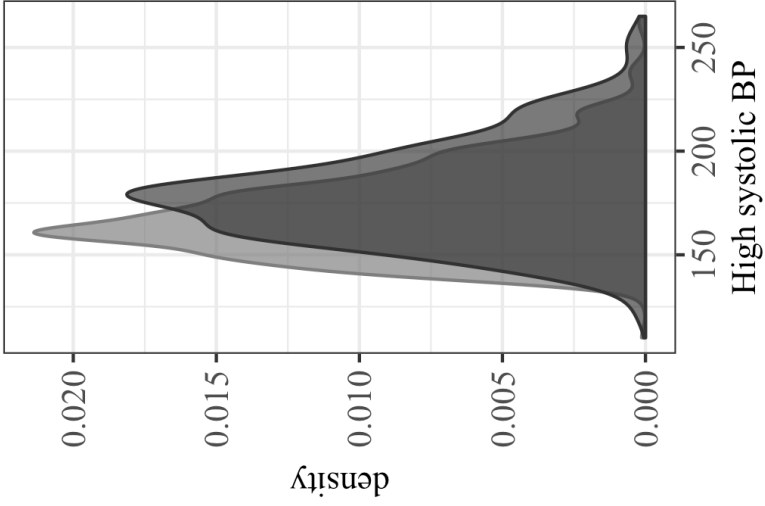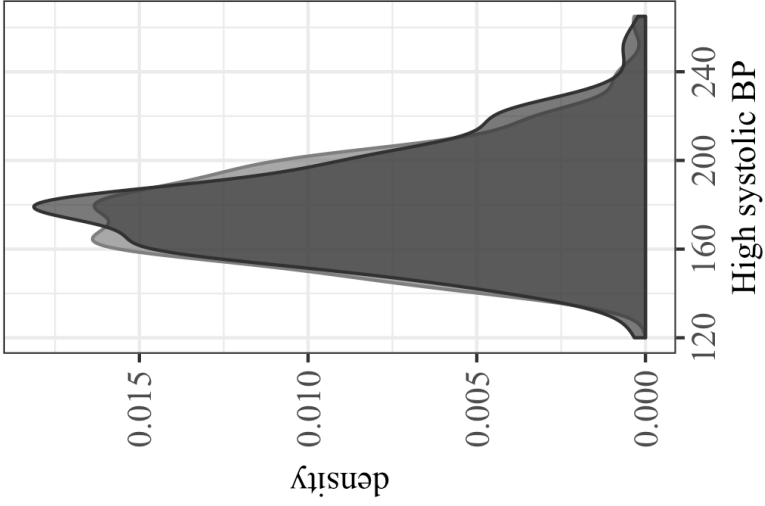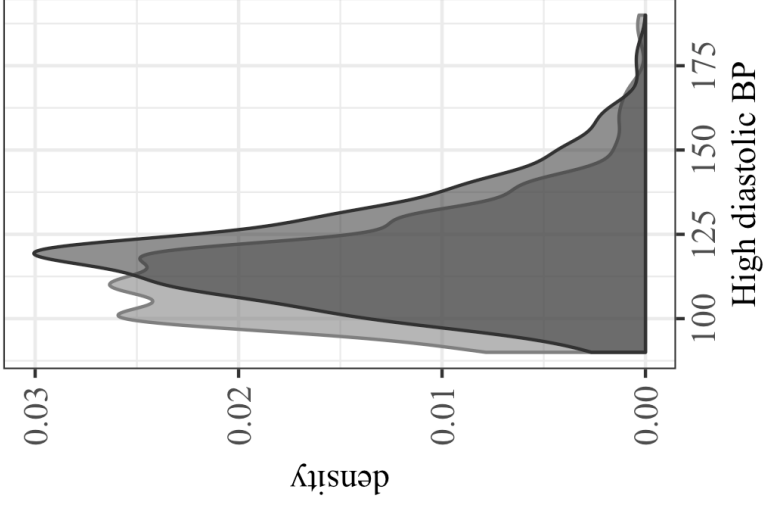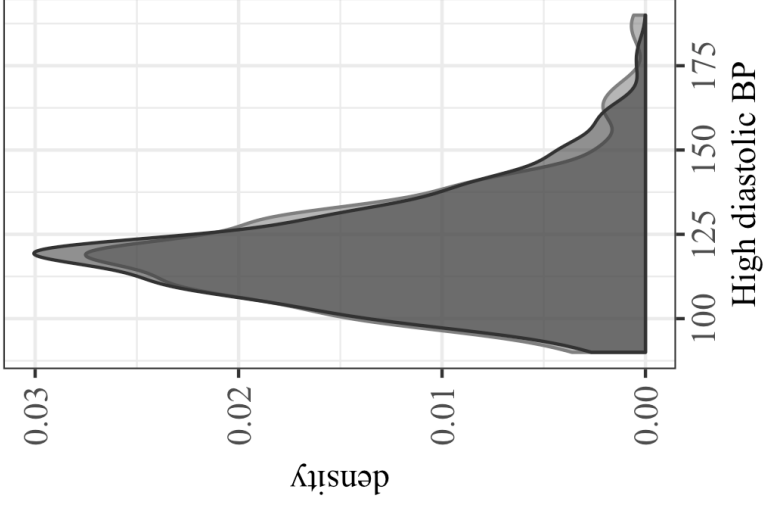

Supplement: Supplementary data 6 [file mmc6.pdf]

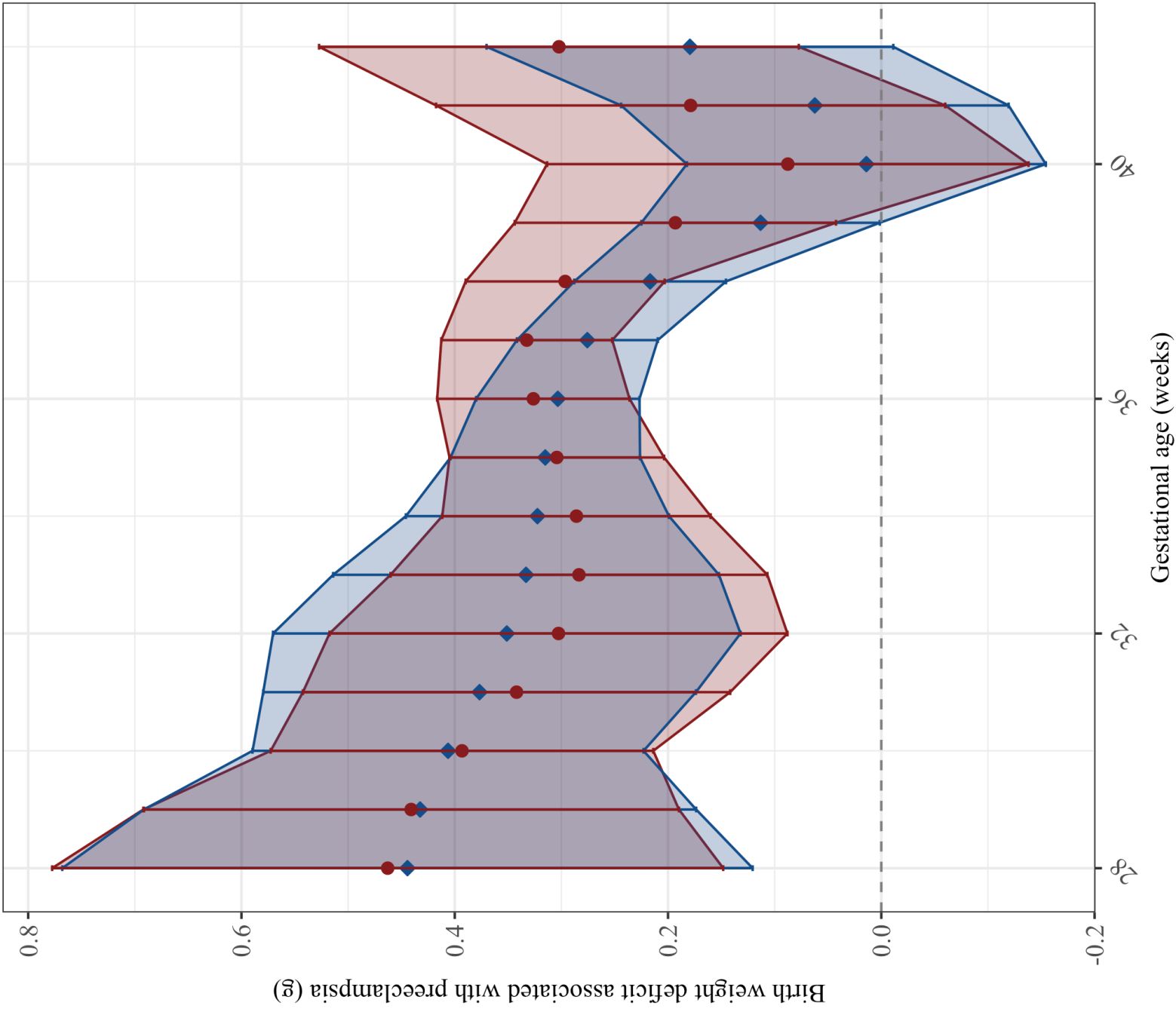

Dataset

- Matched
- Original

Supplement: Supplementary data 7 [file mmc7.pdf]

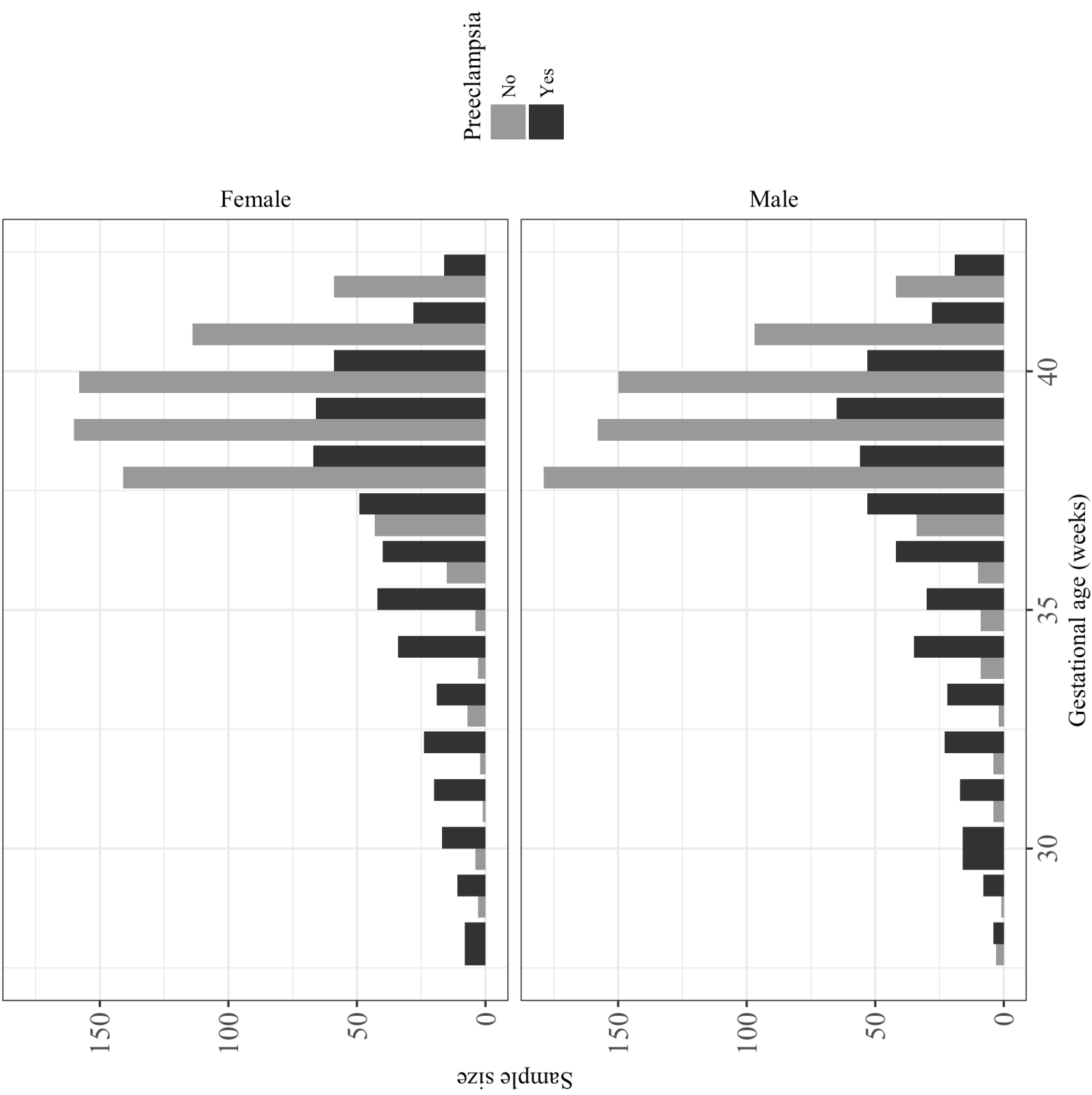

Supplement: Supplementary data 8 [file mmc8.pdf]

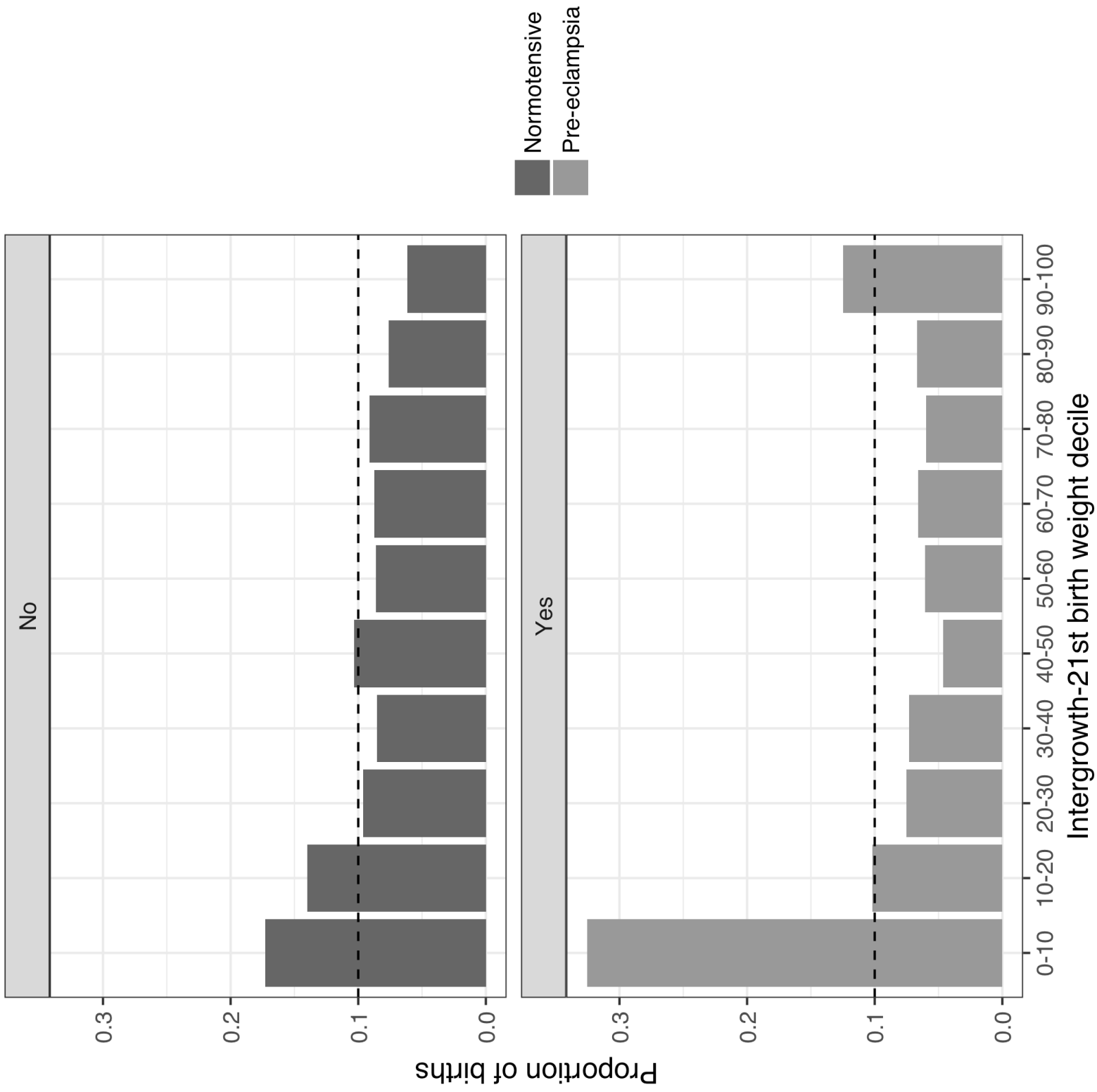

Supplement: Supplementary data 9 [file mmc9.pdf]
